# An Unusual Source of Lead Poisoning

**Published:** 1911-03-25

**Authors:** 


### Toxicology.

### AN UNUSUAL SOURCE OF LEAD POISONING.

The difficulty of tracing the source from which plumbic patients, other than those who are plumbers, painters, or workers at occupations in which lead salts are known to be employed, have acquired the poison is often very great, and the toxic effects may slowly accumulate for many years without the source of danger being recognised at all, as in a recent legal case connected with soda-water syphons. The way in which leakage from an electric main may sometimes cause lead to contaminate the water-supply of a single house in a street as the result of some process of electrolysis of the pipes through which the water flows, has been demonstrated before now.

A still more unusual source of the poisoning has recently occurred. It was noticed that a number of workmen within a limited area complained of obscure attacks of abdominal pain of a colicky nature, associated with troublesome constipation, and in one or two cases, though by no means in all, with a blue line upon the gums. It should be remembered that the presence of a blue line is by no means proof that other symptoms in the case are plumbic; and also that the absence of a blue line upon the gums by no means excludes the possibility of the patient being a sufferer from lead poisoning. When a careful investigation into the cases of these workmen was made, it was found that the only obvious point common to them all was that they obtained their meals in the middle of the day at the same eating-house. Naturally, it was supposed that the drinks were the source of infection; but upon further investigations this could not be confirmed.

The eating-house keeper was indignant at the suggestion that any ill-effects at all were due to food or drinks that he was supplying. So clearly did it appear, however, that the plumbic symptoms followed the taking of meals at this place that even more minute inquiries were made, and the keeper of the eating-house was asked whether he himself or any of his family had ever suffered from similar symptoms. He denied that any of his family were thus affected, until it suddenly occurred to him that his cook had often complained recently of acute abdominal pains of a colicky nature. This at once suggested that it was through the food rather than through the drinks that the lead was getting into the alimentary canals of the different patients. Further inquiries were made, and it was ascertained that upon the demolition of an old house in the neighbourhood a large quantity of timber thickly coated with paint had been purchased by the keeper of the eating-house, and used by him upon the fire upon which the cook prepared food for the workmen. It seemed that the burning of the wood had volatilised enough lead from the paint to contaminate the food that was being cooked, and thus produce lead poisoning in a considerable number of individuals.

